# Thermal Behavior Improvement of Fortified Commercial Avocado (*Persea americana* Mill.) Oil with Maqui (*Aristotelia chilensis*) Leaf Extracts

**DOI:** 10.3390/antiox10050664

**Published:** 2021-04-24

**Authors:** Marcos Flores, Luis Reyes-García, Jaime Ortiz-Viedma, Nalda Romero, Yesica Vilcanqui, Cristian Rogel, Javier Echeverría, Oscar Forero-Doria

**Affiliations:** 1 Departamento de Ciencias Básicas, Facultad de Ciencias, Universidad Santo Tomás, Talca 3460000, Chile; luisreyesga@santotomas.cl; 2 Departamento de Ciencia de los Alimentos y Tecnología Química, Facultad de Ciencias Químicas y Farmacéuticas, Universidad de Chile, Casilla 233, Santiago 8320000, Chile; jaortiz@uchile.cl (J.O.-V.); nromero@uchile.cl (N.R.); 3 Escuela de Ingeniería Agroindustrial, Universidad Nacional de Moquegua, Prolongación Calle Ancash S/N, Moquegua 18001, Peru; yvilcanquic@unam.edu.pe; 4 Departamento de Ciencia y Tecnología de los Alimentos, Facultad de Farmacia, Universidad de Concepción, Concepción 4030000, Chile; crogel@udec.cl; 5 Departamento de Ciencias del Ambiente, Facultad de Química y Biología Universidad Santiago de Chile, Santiago 9170022, Chile; javier.echeverriam@usach.cl

**Keywords:** avocado oil, thermo-oxidative stability, maqui leaf extracts, polar compounds, antioxidant activity

## Abstract

Avocado oil is considered a highly prized food due to its nutritional contribution. On the other hand, *Aristotelia chilensis* (Molina) Stuntz (*Elaeocarpaceae*), common name “maqui”, is an endemic fruit in Chile, well known for its exceptional antioxidant properties. In general, maqui by-products such as leaves are considered as waste. Thus, maqui leaves extracts were used to improve the stability of vegetable oils, particularly avocado oil. Hence, avocado oil was fortified with two extracts (ethyl ether and methanol) obtained of maqui leaves and exposed to 120 °C for 386 h in an oven. The results showed a high content of monounsaturated fatty acids (69.46%, mainly oleic acid), followed by polyunsaturated fatty acids (16.41%, mainly linoleic acid) and finally saturated fatty acids (14.13%). The concentration of the total phenolic compounds in the pure oil, ethyl ether and methanol maqui leaves extracts were 45.8, 83.7, and 4100.9 ppm, respectively. In addition, the antioxidant activity was 5091.6 and 19,452.5 µmol Trolox eq/g for the ethyl ether and methanol extracts, respectively. The secondary degradation compounds showed significant differences between the fortified and non-fortified samples after 144 h and the TG/DTG analysis showed a significant increment of 7 °C in the degradation temperature (Tonset) of avocado oil fortified with the methanol extract when compared to the non-fortified oil and fortified oil with ethyl ether extract. After heating for 336 h, fortified oil with methanol extract reached the limit percentages of polar compounds, while pure oil reached it in a shorter time, i.e., 240 h. Based on the results, avocado oil can be protected with natural additives such as extracts obtained from maqui leaves, leading to an increase in its thermo-oxidative stability.

## 1. Introduction

The use and consumption of avocado oil has increased in the last decade due to the positive effects in areas such as health, cosmetics, and technology [1]. Avocado oil has a high content in oleic acid as well as a low content in saturated fatty acids. For this reason, it is preferred in diets to reduce cardiovascular diseases [2]. Avocado oil is characterized by a low concentration of saturated fatty acids (10–19%), which depends on its state of maturity, variety, and geographical origin [3,4]. In addition, it has a high concentration of oleic acid (>80%) and an acceptable level of polyunsaturated fatty acids (11–15%) [2,5]. Moreover, avocado oil has a high concentration of β-sitosterol, vitamin E, α-tocopherol, and low amounts of squalene, aliphatic, and terpenic alcohols, and other unsaponifiable compounds with biological activity [6]. Unlike industrially refined vegetable oils which are fortified with synthetic antioxidants such as hydroxybutylanisol (BHA), butylhydroxytoluene (BHT) and tert-butylhydroquinone (TBHQ), are usually added to extend the shelf life of edible oils [7,8]. Avocado oil is marketed mainly as an unrefined oil, avoiding the use of synthetic components, which are often questioned due to possible negative health effects [1,9]. However, due to lipid deterioration processes such as thermo-oxidation, it is necessary to find safe, effective, and innocuous antioxidants.

In the field of Food Science, an important challenge is to improve the shelf life of edible oils, due to the susceptibility to oxidation, especially when edible oils undergo heating processes. So far, there has been little interest in the thermo-oxidative behavior of avocado oil in different deterioration conditions [10,11,12]. Therefore, the study and use of plant by-products or natural extracts to improve the stability of edible oils or lipid matrices, to avoid using synthetic antioxidants, is becoming increasingly stronger. For instance, the following plants extracts have been used to improve the stability of edibles oils: Rosehip extracts to improve the thermo-oxidative deterioration of grapeseed oil; methanolic extracts of barley seeds to improve the stability of sunflower oil; and carotenoids obtained from dried tomato residues were applied during the thermo-oxidation of different edible oils [13,14,15].

However, there is a lack of research related to the possible effect of native species coming from South America such as *Aristotelia chilensis* (Molina) Stuntz (*Elaeocarpaceae*), common name “maqui”, on the thermo-oxidative stability of edible oils such as avocado oil. Maqui is known worldwide for its extraordinary antioxidant properties found mainly in the fruit [16]. Recently, some benefits and applications of the maqui fruit have been reported, e.g., beneficial postprandial effects after the consumption of the maqui fruit in healthy individuals, antioxidant and antibacterial effects of the maqui fruit present in coatings for food use, preparation of microparticles from the juice of the maqui fruit, among other applications [17,18,19]. On the other hand, it has been discovered that the leaves of the maqui berry have important analgesic, anti-inflammatory, antioxidant [20], antiviral [21], and α-glucosidase inhibitory activities [16].

To our knowledge, there is no information available about the use of maqui by-products such as maqui leaves and their use as an additive in edible oils [22]. Thus, the main objective of this research is to study the thermo-oxidative behavior of fortified avocado oil with two maqui leaves extracts.

## 2. Materials and Methods

### 2.1. Reagents

The Folin–Ciocalteu reagent, gallic acid, phosphate buffer solution, Trolox, standard fatty acid methyl esters (FAMEs), and other chemicals (analytical grade) were purchased from Merck (Santiago, Chile).

### 2.2. Plant Materials

Extra virgin avocado oil was purchased from a local store. *Aristotelia chilensis* (Molina) Stuntz (*Elaeocarpaceae*) leaves of at least 8 cm in length were collected from wild trees located in the Maule Valley, Maule Region of Chile (35°25′36″ S 71°39′58″ W). Maqui leaves were selected by simple random sampling, where three samples of at least 2 kg were obtained. Then, maqui leaves were reduced in sample size by quartering. About 1000 g of *A. chilensis* leaves were air-dried in an oven dryer model UF-55 (Memmert, Schwabach, Germany) at 50 °C for 24 h. Then, they were ground to obtain a powder that passes through a 2 mm sieve, packed in polyethylene bags coated with aluminum, and stored under ambient conditions for later analysis and use.

### 2.3. Preparation of Extracts from Maqui Leaves

Twenty grams of ground maqui leaves (10 mesh size) were placed in a 250 mL flask and mixed with 150 mL of 100% methanol and 150 mL of ethyl ether. The extraction was performed on an orbital shaker model NB-101M, (N-Biotek, Bucheon, Korea) for 48 h under ambient conditions (stirring intensity 200 rpm). Then, the extraction solution was filtered through a filter paper (Whatman No 1). Next, the solid residues were re-extracted twice with 150 mL of new solvent (i.e., methanol and ethyl ether) each time and the extracts were pooled. The methanol (M) and ethyl ether (E) extracts of maqui leaves were concentrated to dryness under reduced pressure at 45 °C, using a rotary evaporator.

### 2.4. Thermal Analysis

Oil analysis for enriched and non-enriched samples was performed on a TGA-Q500 thermogravimetric analyzer (TA-instruments, Hertfordshire, UK). The temperature range for this study was from room temperature to 700 °C. The furnace heating rate was 5 °C·min^−1^ with a controlled mass flow of air of 60·mL min^−1^ as reagent gas. Additionally, N_2_ with a flow of 40 mL·min^−1^ was used as protective gas. The sample mass placed in a platinum crucible (Pt) inside the balance was around 10 mg for each analysis. Subsequently, the thermogravimetry (TG) and differential thermogravimetry (DTG) curves were analyzed.

### 2.5. Thermooxidation Test and Sampling Period

Four hundred grams of pure, unfortified avocado oil (OP), avocado oil enriched with methanol extract of maqui leaves (OM), and avocado oil enriched with ethyl ether extract of maqui leaves (OE) were added to different 500-mL glass beakers and heated at 120 ± 1 °C in the oven (UF-55) for 386 h. Fortified oils were prepared at a concentration of 800 ppm based on an oxidative stability study of different additives on lipid matrices [23]. Samples were collected and analyzed every 72 h until reaching 386 total hours. Analyses were conducted in triplicate.

### 2.6. Analytical Determinations

#### 2.6.1. Total Phenolic Content (TPC)

The total phenolic content was determined by the Folin–Ciocalteu reagent according to the method proposed by Chun et al. (2005) with minor modifications [24]. The absorbance was measured at 765 nm in a UV/VIS UV3-200 model ATI Unicam spectrophotometer (UNICAM, Cambridge, UK). Gallic acid solutions were used for the calibration curve with six points. The results were expressed in mg GAE/kg of sample.

#### 2.6.2. Antioxidant Capacity: ORAC Assay

The antioxidant capacity of the samples was carried out as described by Dávalos et al. (2004) with some modification [25]. A 0.075M phosphate buffer solution (pH 7.0) was prepared, then a stock solution of fluorescein was prepared by dissolving 0.0038 g of fluorescein in 10 mL of buffer (solution 1), a second stock solution was prepared by diluting 100 µL in 1 mL of buffer (solution 2), and 100 µL of solution 2 was added to 10 mL of phosphate buffer (solution 3). A Trolox 1 mM standard was prepared, and from the Trolox stock solution, an aliquot of 50 µL was taken. In 450 µL of phosphate buffer, this solution was divided into aliquots in small vials to perform the Trolox calibration curve (10, 20, 30, 40, 50, 60 µM) in phosphate buffer in appropriate wells. For the analyses, 96-well black microplates were used in which excitation (485 nm)/emission (538 nm) was produced from the top of the dish. The buffer solution was utilized as a blank to dilute the samples. To perform the ORAC analysis, 25 µL of the diluted sample containing the phenolic compounds, 150 µL of fluorescein were added to the 96-well black plate, the microplate was incubated for 30 min at 37 °C, and then the reaction was initiated by the addition of 25 µL (75 mM) of AAPH (2,2′-Azobis (2-methyl-propionamidine) dihydrochloride). Readings were obtained from a microplate reader model synergy HTX (Biotek, Winooski, VT, USA). The antioxidant capacity was expressed as µMeq Trolox/g of sample.

#### 2.6.3. Gas Chromatography Coupled with Flame Ionization Detector (GC/FID)

Fatty acid profile was obtained for avocado oil by using fatty acid methyl esters derivatives and analyzed by gas chromatography coupled to a flame ionization detector (Hewlett-Packard, Palo Alto, CA, USA), equipped with a split-splitless injector. The separation of the different fatty acid methyl esters was done in a capillary column BPX-70 (50 m length, 0.32 mm internal diameter, 0.25 μm film thickness; SGE, Melbourne, Australia). The carrier gas was hydrogen at a flow rate of 1 mL·min^−1^. The conditions for the oven were as follows: The initial temperature was set to 160 °C with a temperature gradient from 180 to 230 °C at 2 °C·min ^−1^. The sample size was 1 µL. The identification of the fatty acids was done by comparison of their retention times with those of standard fatty acid methyl esters (FAME) from Merck (Merck, Darmstadt, Germany) [26].

### 2.7. Specific Extinction Coefficient (k_270_ and k_232_) of Avocado Oil

The extinction coefficient (*k*_270_ and *k*_232_) was determined following the protocol proposed by Antolin and Meneses (2000) with minor modifications [27]. Specifically, avocado oil samples were diluted in n-hexane to obtain a 1% (*w*/*v*) solution. Then, the oil samples were measured using quartz cuvettes with pure solvent as a reference. Absorption measures were taken at *k*_232_ and *k*_270_ nm in a UV spectrophotometer (PG Instruments T80 + UV-visible spectrometers). The values of k were calculated according to Equation (1):

kλ = Abs λ/D × L(1)

where Abs λ is the absorption, D is the dilution expressed in g/100 mL; L is the cuvette pathlength (1 cm path); and kλ is the specific extinction coefficient at different wavelengths.

### 2.8. Calculated Oxidizability Value (Cox Value)

The Cox value of the oil was calculated based on the sum of the percentages of unsaturated fatty acids multiplied by proportionality factors, applying the formula proposed by Fatemi and Hammond (1980) [28].

Cox value = [1 × (16:1% + 18:1%) + 10.3 × (18:2%) + 21.6 × (18:3%)]/100(2)

### 2.9. Total Polar Compounds

The percentage of polar compounds was determined with the use of an electrochemical sensor model Testo 270 (Testo AG, Lenzkirch, Germany). The instrument conditions were those indicated by the manufacturer. Briefly, once the oil samples were removed from the oven to carry out the different physical-chemical analyses (around 30 mL), the testo sensor was immediately introduced into the solution, respecting the time required for a stabilized reading. Polar compounds were expressed as percentages.

### 2.10. Statistical Analysis

A one-way ANOVA was performed to determine if there were statistically significant differences between the samples (fortified and non-fortified oils). The comparison of means was made using the least significant difference (LSD) method using IBM SPSS Statistics Version 19 (International Business Machines Co., Armonk, NY, USA). The differences between samples were considered significant for a confidence interval at the 95% level (*p* < 0.05) in all cases.

## 3. Results and Discussion

Table 1 shows the fatty acid profile of avocado oil, which is mainly monounsaturated fatty acids (oleic acid > 65%) followed by a lower proportion of polyunsaturated fatty acids (16.41%) and saturated fatty acids (14.13%). Thus, avocado oil has a polyunsaturated/saturated ratio (P/S) equal to 1.16 and a Cox value of 2.53. These values demonstrate the oxidation susceptibility of avocado oil, which is slightly higher than those for other vegetable oils such as olive oil [29]

In general, edible oils used in food are rich in unsaturated fatty acids, mainly linoleic acid from corn [30], soybean [31], and sunflower [31], and oleic acid from canola (60.7%) [32], avocado (65.3%) [10], and olive (66.4–81.5%) [33,34]. Non-conventional oil sources such as jerivá [35], araça [36], and coffee (Arabica and Robusta varieties) [37,38] are also predominantly high in unsaturated fatty acids.

The fatty acid profile of avocado oil reported in previous studies shows a traditional composition range from highest to lowest proportion in the following order; oleic (C18:1) 60–80%, palmitic (C16:0) 10–25%, linoleic (C18:2) 7–20%, palmitoleic (C16:1) 2–8%, stearic (C18:0) 0.1–1.5%, and linolenic (C18:3) 0.2–1.0% acids [39,40]. The differences in linolenic acid composition from the avocado oil obtained from Chile with other geographical regions were ∆C18:3 = 0,26. The percentages of linolenic and linoleic acids decrease at higher temperatures. This decrease is accompanied by an increase in oleic acid [41].

Recently, others range have been reported for fatty acids from several avocado oils, which include increasing the proportion of unsaturated fatty acids, and therefore their predisposition to oxidation: Palmitic at 10.0–35.2%; palmitoleic at 2.8–16.1%; stearic at 0.2–1.5%; oleic at 36.9–74%; linoleic at 6.1–21.2%; and linolenic at 0.3–2.1%. In addition, the presence of ω7, ω9, and ω11 isomers of oleic and palmitoleic acids in avocado oil has been described [42]. During the oxidation of edible oils at high temperatures (>100 °C), it has been shown that polyunsaturated fatty acids have a higher degradation rate, followed by monounsaturated fatty acids and finally saturated fatty acids [43]. On the other hand, compounds found in edible oils, such as tocopherols and tocotrienols, decrease dramatically as deterioration is prolonged (<30% at the initial concentration) [13]. It is essential to know the proportion of each group of compounds and the relationship between them because it will define the oxidation susceptibility of the edible oil. For example, the P/S ratio is related to the oxidation susceptibility. In this case, the value presented by the avocado oil (1.20) is similar to Chilean hazelnut oil (1.16), which has been shown to have an adequate thermal stability [44].

**Figure 1:**
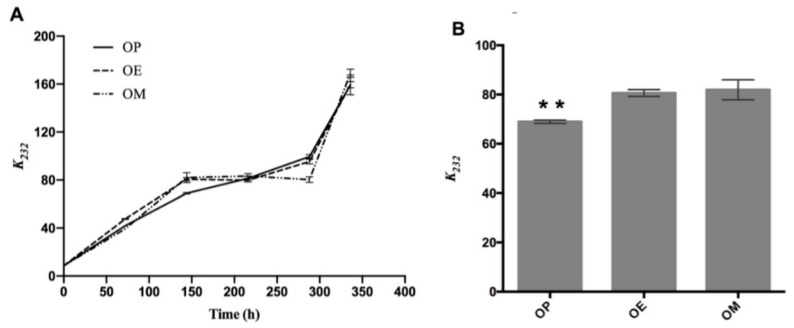
Evolution of primary oxidation compounds during the thermo-oxidation of avocado oil (OP), avocado oil fortified with methanolic (OM), and ethyl ether (OE) maqui extracts. (**A**) evolution of *k*_232_ as time increases, (**B**) statistical analysis of the *k*_232_ value at 144 h. OP: avocado oil. ** *p* < 0.01 with respect to OE and OM oils.

In Figure 1A, the evolution of the absorption coefficient *k_232_* of the OP, OM, and OE samples exposed to 120 °C for 336 h is shown. In general, an increase in this parameter is observed in all samples as the heating time increases. Significant differences of the *k_232_* value at 144 h of OP (68.95 ± 0.67, ** *p* < 0.01), with respect to OE and OM oils, are observed (Figure 1B).

The same trend has been documented during the thermo-oxidation of different edible oils. It has been correlated with an increase in the concentration of primary oxidation compounds and therefore the quality of the oils [27]. The curves for OP, OM, and OE showed no significant differences. Therefore, the oil fortified with the OM and OE, respectively, did not have a protective effect against the formation of primary oxidation products (namely, peroxides and hydroperoxides with conjugated double bonds) compared to OP. Primary oxidation products are known to be unstable during thermo-oxidative deterioration. These by-products are rapidly transformed into secondary oxidation products such as aldehydes, ketones, and alcohols [45].

**Figure 2:**
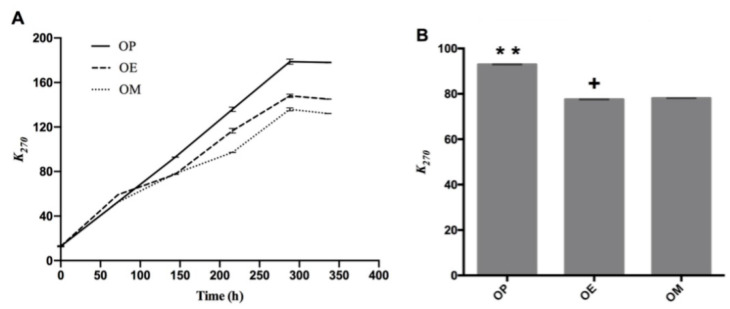
Secondary oxidation compounds during the thermo-oxidation of avocado oil (OP), avocado oil fortified with methanolic (OM) and ethyl ether (OE) maqui extracts. (**A**) evolution of *k*_270_ as time increases, (**B**) statistical analysis of the *k*_270_ value at 144 and 336 h. OP: avocado oil. ** *p* < 0.01, with respect to OE and OM oils and + *p* < 0.05 of OE with respect to OP and OM.

Regarding the absorption coefficient *k*_270_, which is shown in Figure 2A, a separation of the curves (OP, OE, and OM) can be observed after heating for 140 h, with a significant difference of the values of *k*_270_ (*p* < 0.05; *p* = 5.8 × 10^−12^) at 144 h. The OP oil showed the highest *k*_270_ value with significant differences at 144 h (92.96 ± 0.14, ** *p* < 0.01), while OE and OM oils showed *k*_270_ value of 77.55 ± 0.13 (*p* < 0.05) and 78.09 ± 0.05 (Figure 2B), respectively. On the other hand, OP showed significant differences at 336 h (178.03 ± 0.42, **** *p* < 0.0001) with respect to OE and OM with *k*_270_ values of 145.08 ± 0.18 and 132.16 ± 0.15, respectively. As is observed, there is an approximate decrease of 18 and 26% of *k*_270_ values of OE and OM, making the methanol extract of maqui leaves (M) a better antioxidant.

This increase can be explained by the presence of oxygen in the oven due to the wide contact area (52.8 cm^2^) between the lipid mass and the air mass above the oils. On the other hand, ethyl ether (E) and methanol (M) extracts of maqui leaves exert an antioxidant effect in the avocado oil reducing the *k*_270_ value in 15.41 and 14.87%, respectively.

In a previous work, samples of commercial avocado oil from Chile showed *k*_270_ values of 0.72 ± 0.01 and 0.17 ± 0.003, and *k*_232_ values of 4.19 ± 0.09 and 3.16 ± 0.06 at 25 °C [40]. Avocado oil samples from Mexico have shown *k*_232_ values in the range between 0.31–1.06, *k*_268_ in the range from 0.017–13, and *k*_274_ between 0.013–0.12 [46] at room temperature. Recently, Resende et al. [47] reported that the *k*_232_ and *k*_270_ values in avocado oil increased linearly at a higher rate, becoming constant or decreasing after a short reaction time at high temperatures.

Our results are in agreement with the observations of Resende et al. (2019) [47] where *k*_232_ and *k*_270_ values increased linearly at a higher rate with increasing time at study temperature (120 °C). The OP curve after 144 h continues with sustained increase and greater than the curves for OE and OM until the end of the heating period (336 h). The results show that the methanol extract had the best performance against the production of secondary oxidation compounds, producing a lower number of secondary compounds after around 100 h of heating.

The protective effect for avocado oil fortified with the ethyl ether extract and the methanol extract can be attributed to the addition of phenolic compounds present in them. According to Table 2, it can be seen that OM has a total phenolic content greater than 40 times than OE. A greater protective effect from OM can be explained by the higher content of total phenols compounds present in the extract due to the affinity of the phenolics compounds with the polarity of the solvent. OM and OE extracts exhibited an antioxidant capacity of 19,452.5 ± 2111.1 and 5091.6 ± 174.6, respectively, expressed as µmol Trolox eq/g dry wt.

Previous studies in samples of two commercial avocado oils from Chile showed a total phenolic content (TPC) of 42.6 ± 1.1 and 56.9 ± 0.9 mg/Kg [40], while samples from South Africa had a total phenolic content of 0.58 ± 0.66 mM TAEC/kg [48]. The sample from Mexico showed a TPC between 0.51–11.77mg/g and ORAC values between 28.24–65.92 µmol/Kg [46].

Jiménez et al. (2017) [49] reported the effects of hydroalcoholic extracts of olive leaf (OHE) on the thermal stability of two mainly monounsaturated vegetable oils such as canola oil (CO) and high oleic sunflower oil (HOSO), both with over 50% monounsaturated fatty acids, during French potatoes frying at 180 °C. The fortification of the extract in both oils decreased the formation of polar compounds, this protective phenomenon can be attributed to the compounds present in the extract such as oleuropein and its derivatives, oleuroside and oleuroside-10-carboxylic acid.

Sun-Waterhouse et al. (2011) [50] investigated the effects of specific phenolic compounds on the thermal stability of avocado and coconut oils at various temperatures with a maximum of 60 °C. The incorporation of phenolic compounds during prolonged storage protects the degradation of unsaturated fatty acids; however, hydrolysis of triacylglycerols has been demonstrated. Other studies demonstrate the nutritional benefits of fortifying avocado oil with encapsulated antioxidant compounds such as floridzin, at medium temperatures such as 37 °C [51]. However, it is necessary to deepen its behavior at high temperatures to bring the research closer, for example, to the cooking and food preparation processes as proposed in this study.

The AC value obtained for the OM is approximately four times greater than OE. These results are related to the higher affinity of the antioxidant compounds for the more polar solvent, which is in accordance with the total phenolic content for each extract. The values reported in this study are higher than those obtained for other wild fruits calculated by the ORAC method, which ranged from 519.2 to 854.8 μmol Trolox Eq/g dry wt. The results may be related to a marked protective effect of extracts from the maqui tree leaves [52]. In the literature, it has been described that extracts from solvent mixtures of high polarity (ethanol/water) of maqui leaves have phenolic components such as phenolic acids, flavonoids, anthocyanins, flavanols, and stilbenes, as well as alkaloids and phytosterols, which also have antioxidant activity [53,54]. In addition, extracts of different polarities of the maqui have shown interesting results for antioxidant activity, antibacterial capacity, and a protective effect in a lipid model due to the phenolic composition of leaves [16,22]

**Figure 3:**
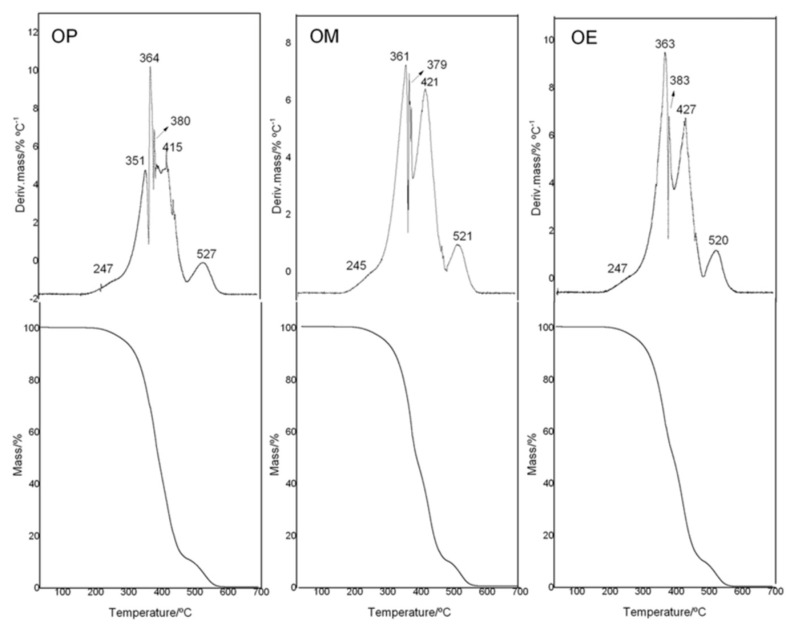
TG/DTG curves of avocado oil (OP), and avocado oil fortified with methanolic (OM) and ethyl ether (OE) extracts of maqui leaves.

To analyze the thermal stability of avocado oil (OP, OM, and OE samples), thermal analyses were done in an interval of 20–700 °C in an oxidant atmosphere. The TG/DTG curves are shown in Figure 3. The observed thermal degradation for OP, OM, and OE occurred in five and six steps of degradation. The TG-plateau of for the OP and OM and OE samples showed differences in the maximum degradation temperatures of the different step-thermal behavior.

The onset temperature of mass loss (Tonset/°C) (Table 3) for OP and OE were similar with a value of 233–234 °C, while OM (Tonset/°C: 240) had an increase of ≈7 °C with respect to OP and OE.

With a mass loss of 5, 10, and 50%, the temperature of degradation for OP oil was found to be at 288, 319, and 389 °C, respectively, while OE presents values of 286, 315, and 391 °C, with a decrease being observed around 2–4 °C at 5 and 10% of mas loss and only an increase of 3 °C at 50% of mass loss for no-enriched OP. On the other hand, OM oil showed high temperature displacements to 6 °C for 5 and 10% of mass loss in comparison with OP oil. Our research group reported similar results as those by a study related to the effect of eugenol as an additive used in essential oils of *Hedychium coronarium* J. Koening (*Zingiberaceae*). The results showed an increase of 12 °C at the supplementation of 4.7% (*v*/*v*) for T20%/°C [55]. The ability to enhance the thermal stability of the methanol extract from maqui leaves in avocado oil is no surprise since that OM is fifty and four times greater than that of the OE as far as its phenol composition and antioxidant activity are concerned (Table 2). These results are related to the higher affinity of the antioxidant compounds for the solvent of greater polarity, which is in accordance with the total phenolic content for each extract.

In relation to the thermal stability of avocado oil, it has recently been reported that the first step of thermal decomposition (I) was observed between 230–286 °C, with the maximum temperature at 308 °C and 38.2% of mass loss. In the second step of decomposition (II), both the avocado oil suffered considerable mass losses (34.3%) [56].

It is important to note that, although OM oil has a higher content of phenolic compounds than OE oil, the thermal behavior was not significant different. A similar trend has been reported in different studies where the effect of phenolic content of different edible oils and the relationship with their thermal behavior have been analyzed [10,11,44]. It is known that phenolic compounds, together with tocopherols (α and β), contribute to the oxidative stability of edible oils [57]. However, the OM oil could be low in tocopherols. Therefore, the presence of other phenolic compounds would explain the antioxidant activity, such as gallic acid, catechin, rutin, and *p*-coumaric acids, among other [50,58].

It has been reported that avocado oils has a predicted shelf life of 210 days at 25 °C [59]. However, its shelf life can be affected by exposure to light and high temperatures.

In order to protect edible oils from oxidation, Jiménez at al. (2017) reported the effects of hydroalcoholic extracts of olive leaves (OHE) on the thermal stability of canola oil (CO) and high oleic sunflower oil (HOSO) of French potatoes frying at 180 °C. The addition of OHE to CO and HOSO decreased the polar compounds formation, which could be attributed to the protective action of polyphenols as oleuropein and its derivatives, oleuroside and oleuroside-10-carboxylic acid [49,60]. Other edible oils, such as coconut and avocado oils, have been fortified with hydroxycinnamic acids such as caffeic (CA) and p-coumaric (pCA) acids. The hydroxycinnamic fortification (300 ppm; premixed with PEG (3%, *w*/*w*)) of both oils were stored at 20 and 60 °C for 50 days, and oxidation parameters were monitored, namely, peroxide values, p-anisidine value, and free fatty acids, among other. The results showed that the oxidation process was accelerated when samples were stored at 60 °C. However, CA and pCA helped preserve avocado and coconut oils from oxidation [50]. Similar results have been described for avocado oil fortified with phlorizin (dihydrochalcone) encapsulated with alginate, in the study for improving the oxidative stability and suppressing hydrolytic rancidity of avocado oil at 37 °C [51].

Despite the possible differences in the composition of phenolic compounds in OE and OM oil previously described, the difference of LogP and ESOL solubility of the different phenolic compounds (Table 4) play an important role in the triglyceride hydrophobic interaction of phenolic compounds and avocado oil. The hydrophobic interaction of α and β-tocopherol (LogP: 8.27 (α); 7.79 (β)) in OE was greater than that shown by gallic acid (LogP: 0.21), catechin (LogP: 0.83), rutin (LogP: -1.12), and *p*-cumaric acid (LogP: 1.26) in OM.

This would explain that even though the number of phenolic compounds in EO is fifty times lower than in OM, Tocopherols present in OE had a higher LogP values than phenols contained in OM, maintaining good protection.

The results of this last analysis are corroborated by the index of polar compounds. This index is considered a robust indicator to describe the deterioration of fatty material because it involves the degradation products of triacylglyceride.

**Figure 4:**
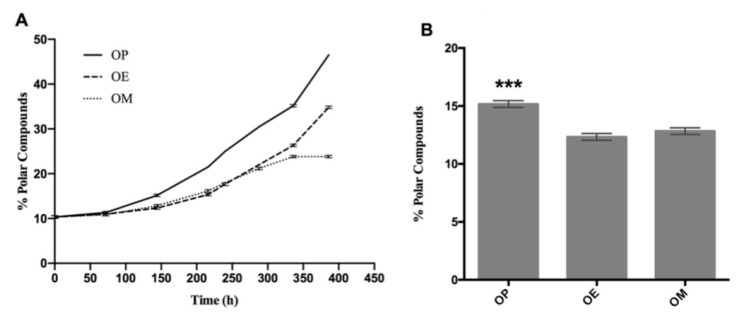
Evolution of total polar compounds during the thermo-oxidation of avocado oil (OP), avocado oil fortified with methanol (OM) and ethyl ether (OE) extract of maqui leaves. (**A**) polar content as time increases, (**B**) statistical analysis of the polar content at 144 and 336 h. OP: avocado oil. *** *p* < 0.001, with respect to OE and OM oils.

According to Figure 4A, the index of polar compounds is increasing for the three curves (OP, OE, and OM). However, after heating 144 h, there is a significant difference in the content of polar compounds. Thus, the OP oil showed a percentage of polar compounds with significant differences at 144 h (15.16 ± 0.29, *** *p* < 0.001), with respect to OE and OM oils (Figure 4B).

Overall, OP continues with a sustained increase above the OE and OM curves. Finally, the OE and OM curves show a separation at 288 h (*p* < 0.05; *p* = 0.01). On the other hand, OP continues with an increase rate above OE and OM, with significant differences at 336 h (35.20 ± 0.29, **** *p* < 0,0001) with respect to OE and OM with %polar compounds of 26.3 ± 0.29 and 23.8 ± 0.29, respectively. A decrease of 25 and 32% was observed in the OE and OM oils, respectively, with the methanol extract of maqui leaves being more effective in antioxidant protection. This trending is observed across the different oxidation level measurements performed. Finally, OP and OE continue to increase until the end of the experiment (386 h), while OM maintains constant polar compounds levels.

The index of polar compounds is recognized as a parameter for quality control in different regulations in the world. In this case, it indicates that an oil destined for a thermo-oxidative process, such as the deep fat frying process, cannot exceed a value of 25% [61]. Thus, the OP, OE, and OM samples reached this value at approximately 250, 310, and 360 h after heating, respectively, which demonstrates the protective effect of maqui leaves extracts.

According to this study, there is a lower production of lipid degradation compounds, when the extracts of maqui leaves are present. This protective effect could benefit other oils of great interest to the health of the population due to their high PUFA content, with oils such as grapeseed oil (66–75% in linoleic acid) obtained from by-products of the wine industry, of great nutritional interest. Grapeseed oil has been related to anti-inflammatory properties, cardio-protective, antimicrobial, and anticancer [62]. Additionally, there are other oils with a high PUFA content along with other components of nutritional interest, such as cotton, chia, or sesame oils, which could benefit from the antioxidant fortification from natural extracts [63,64]. However, more studies are needed to elucidate the possible protective effects of native plant extracts.

## 4. Conclusions

Avocado oil has different fatty acids and naturally occurring antioxidant components that protect the oil against thermo-oxidative deterioration. Fortification with maqui leaves extracts using two solvent improves the thermo-oxidative stability of the pure oil, demonstrating a beneficial effect for avocado oil. The methanolic extract has a better protective effect on the thermo-oxidation of the oil after heating for 336 h. In addition, secondary oxidation products demonstrate a marked difference between the fortified and non-fortified samples after 144 h of heating. By using solvents of different polarity, the antioxidant properties of plant extracts of *A. chilensis* leaves can be improved. Despite the maqui leaf extract’s well-known and verified positive effect on health, new in vitro and in vivo studies, focused mainly on the toxicity and hormetic role of the antioxidant compounds of maqui leaves, should be carried out in the future before its secure application as a food additive. Finally, the use of by-products from native plants, such as leaves, can be an alternative to the addition of non-natural compounds in oils.

## Figures and Tables

**Table 1:** Fatty acids profile of avocado oil.

Fatty Acid	Percentage (%)
C14:0	0.054 ± 0.001
C16:0	13.512 ± 0.001
C16:1 ω 9 ω 7	5.398 ± 0.004
C18:0	0.474 ± 0.001
C18:1 ω 9	55.954 ± 0.056
C18:1 ω 7	7.222 ± 0.060
C18:2	16.127 ± 0.012
C20:0	0.062 ± 0.002
C18:3	0.935 ± 0.003
C20:1	0.170 ± 0.003
C22:0	0.031 ± 0.002
C24:0	0.056 ± 0.004
Total Saturated	14.190
Total Monounsaturated	68.747
Total Polyunsaturated	17.062
Polyunsaturated/Saturated (P/S)	1.202
Unsaturated/Saturated (U/S)	6.047

**Table 2:** Total phenolic content (TPC) and antiradical capacity (AC) of OP, OE, and OM samples.

Sample	OP	OE	OM
TPC (ppm GAE)	45.8 ± 3.9	83.7 ± 9.8	4100.9 ± 212.6
AC (µmol Trolox eq/g dry wt.)	nd	5091.6 ± 174.7	19,452.5 ± 2111.1

nd. No determined.

**Table 3:** Thermal decomposition temperatures of OP, OM, and OE avocado oils.

Oil	Temperature (°C) Corresponding to			
	*T*_onset_/°C ^a^	*T*_5_/°C ^b^	*T*_10_/°C ^c^	*T*_50_/°C ^d^
OP	234	288	319	389
OE	233	286	315	391
OM	240	292	321	386

Decomposition temperature (°C): ^a^ onset temperature, ^b^ at 5% mass loss, ^c^ at 10% mass loss, ^d^ at 50% mass loss.

**Table 4:** LogP and estimating aqueous solubility directly from the molecular structure (ESOL) from phenols compounds present in maqui leaves.

Compound	MW	Consensus LogP ^a^	ESOL Log S ^b^	ESOL Solubility (mol/L) ^c^	ESOL Class
*α* -tocopherol	430.71	8.27	−8.60	2.50 × 10^−9^	Poorly soluble
*β*-tocopherol	416.68	7.79	−8.29	5.16 × 10^−9^	Poorly soluble
Gallic acid	170.12	0.21	−1.64	2.29 × 10^−2^	Very soluble
Catechin	290.27	0.83	−2.22	5.98 × 10^−3^	Soluble
Rutin	610.52	−1.12	−3.30	5.05 × 10^−4^	Soluble
*p*-cumaric acid	164.16	1.26	−2.02	9.65 × 10^−3^	Soluble

^a,b,c^ LogP and ESOL parameters were computed using the freely accessible web server SwissADME: http://swissadme.ch/index.php# (accessed on 20 January 2021) undefined.

## Data Availability

The data presented in this study are available on request from the corresponding author.
